# Broad-Spectrum Multi-Epitope Design Targeting Conserved Hantavirus Glycoproteins (Gn/Gc): Chimeric Antigen Engineering and Structural Mapping

**DOI:** 10.3390/ijms27157021

**Published:** 2026-08-05

**Authors:** Silvia da Silva Fontes, Fernando Paiva Conte, Jorlan Fernandes, Elba Regina Sampaio de Lemos, Josué da Costa Lima-Junior, Renata Carvalho de Oliveira, Rodrigo Nunes Rodrigues-da-Silva

**Affiliations:** 1 Laboratório de Hantaviroses e Rickettsioses, Instituto Oswaldo Cruz, Oswaldo Cruz Foundation, Rio de Janeiro 21040-900, Brazil; 2 Pilot Plant Implantation Project, Immunobiological Technology Institute, Oswaldo Cruz Foundation, Rio de Janeiro 21040-900, Brazil; 3 Laboratory of Immunoparasitology, Oswaldo Cruz Institute, Oswaldo Cruz Foundation, Rio de Janeiro 21040-900, Brazil

**Keywords:** multi-epitope vaccine, immunoinformatics, hantavirus, broad-spectrum vaccines, hemorrhagic fever with renal syndrome, hantavirus pulmonary syndrome, computational vaccine design

## Abstract

Hantaviruses, the etiological agents of hemorrhagic fever with renal syndrome (HFRS) and hantavirus pulmonary syndrome (HPS), represent a high-risk zoonotic threat with substantial global health impact. Currently, there is no FDA-approved vaccine. The viral surface glycoprotein (GP) is crucial for host cell entry and is regarded as a key target for vaccine development. However, its variability among hantavirus species limits the effectiveness of conventional vaccine strategies. Epitope-based vaccines offer a promising alternative by enabling the design of broadly protective constructs. In this study, we applied immunoinformatics approaches to design a universal multi-epitope vaccine candidate targeting both HFRS- and HPS-associated hantaviruses through a multi-layered workflow integrating B-cell and T-cell epitope prediction, antigenicity scoring, IFN-γ induction potential, conservation analysis, and population coverage assessment. Viral GPs from SEOV, PUUV, SNV, and ANDV were analyzed using algorithms for B-cell and T-cell epitope prediction. Predicted epitopes were assessed for allergenicity, toxicity, conservation, and population coverage. Two vaccine constructs incorporating β-defensin or 50S ribosomal protein L7/L12 as adjuvants were assessed for physicochemical properties, structural stability and immunogenic potential. Molecular docking analyses provided exploratory ectodomain-compatibility screening, suggesting potential interactions with TLR4 that require future experimental confirmation. The in silico immune simulations suggested potential robust and long-lasting responses with memory cell persistence exceeding one year. Simulations also indicated balanced humoral and cellular responses, robust antibody production, and long-term memory formation suggestive of durable protective immunity. These findings support the rational design of broad-spectrum multi-epitope vaccines against genetically diverse hantaviruses, offering a rational framework for preclinical development of next-generation universal vaccines against hantavirus-associated diseases.

## 1. Introduction

Hantaviruses represent an emerging global public health threat, affecting thousands of people worldwide [1]. They belong to the order *Bunyavirales* and the family *Hantaviridae*. Human pathogenic hantaviruses were isolated from rodents and belong to the *Orthohantavirus* genus, of which more than 30 species have been described [2,3]. Human infection usually occurs through inhalation of aerosolized viral particles from the excretions of infected rodents [4]. The clinical manifestations of hantavirus infection exhibit variability depending on the specific strain of the virus [4]. The Old-World hantaviruses, such as *Orthohantavirus seoulense* (SEOV), *O. hantanense* (HTNV), *O. puumalaense* (PUUV), and *O. dobravaense* (DOBV), have been predominantly identified in Europe and Asia. These viruses primarily affect the kidneys, leading to hemorrhagic fever with renal syndrome (HFRS), which can result in a case fatality rate of 1 to 15% [2,4]. PUUV and DOBV have been demonstrated to be associated with nephropathia epidemica (NE), a mild form of HFRS [2,4]. In addition, *O. andesense* (ANDV) and *O. sinnombreense* (SNV), which affect the lungs, are the predominant New World hantaviruses, and the main cause of hantavirus pulmonary syndrome (HPS), a condition with a case fatality rate reaching up to 40%, in South and North America, respectively [2,4]. It is noteworthy that ANDV is the sole hantavirus reported to be transmissible from person to person [5].

Hantaviruses are single-stranded, negative-sense, enveloped RNA viruses with an unusual tripartite segmented genome consisting of small (S), medium (M), and large (L) segments [1,6]. The M segment is responsible for the expression of glycoproteins (GPs), Gn and Gc, which play a critical role in viral entry into susceptible cells [7]. These proteins form a grid-like pattern on the viral surface, composed of Gc and Gn heterodimers that mediate viral entry via receptor-mediated endocytosis and endosomal membrane fusion [8]. Previous studies have demonstrated that neutralizing antibodies primarily target epitopes within Gn and Gc glycoproteins, underscoring their relevance as key immunogens for vaccine development [9,10,11].

Currently, there are no licensed preventive vaccines against Hantavirus in Europe or the United States. However, some ongoing clinical trials are attempting to develop DNA vaccines against ANDV (Phase I; NCT03682107) and HTNV/PUUV (Phase IIa; NCT02116205) [1]. A South Korean formalin-inactivated HTNV vaccine called Hantavax^®^ has been marketed for the prevention of HFRS for 30 years and recently showed promising results in a Phase III clinical trial [6]. GPs have been the primary targets in hantavirus vaccine research, including DNA-based vaccines that focus on the M-segment of the viral genome. Nevertheless, the high diversity of these glycoproteins—particularly Gn—across pathogenic hantavirus species poses a significant challenge to vaccine design and may impact protective efficacy [7,12]. In this context, epitope-based vaccines are emerging as a promising approach to developing a universal hantavirus vaccine. These vaccines are composed of immunogens that contain selected B- and T-cell epitopes [13,14,15,16], capable of simultaneously stimulating robust cellular and humoral immune responses. The incorporation of multiple conserved epitopes derived from distinct viral antigens has the potential to elicit broad cross-protective immunity against diverse hantavirus strains [13].

In the present study, immunoinformatic approaches were employed to analyze the glycoproteins (GPs) of four representative hantavirus species: ANDV and SNV, the primary etiological agents of HPS in South and North America, respectively [17]; SEOV, a globally distributed hantavirus associated with HFRS in the Old World; and PUUV, prevalent in Europe and linked to a milder form of HFRS [18]. This analysis aimed to design a universal epitope-based vaccine capable of providing broad protection against both renal and pulmonary clinical syndromes associated with hantavirus disease.

## 2. Results

### 2.1. B Cell Epitope Prediction

Our study identified 36 potential linear B-cell epitopes in the studied proteins. Specifically, nine epitopes were predicted in SNV, eight in ANDV, ten in SEOV, and nine in PUUV. These epitopes each consisted of at least nine amino acid residues and were predicted as epitopes by at least three algorithms. Each predicted epitope’s antigenicity was assessed, leading to the exclusion of 15 sequences that scored below the 0.4 threshold in the VaxiJen analysis. Therefore, as summarized in Table 1, 21 sequences were selected as antigenic and linear B-cell epitopes, 8 in the GP of SNV, 3 of ANDV, 6 of SEOV, and 4 of PUUV. The complete list of predicted and selected B-cell epitopes is presented in Supplementary Table S1.

### 2.2. T-Cell Epitope Prediction

Using TepiTool, we identified 71 sequences predicted to be potential T-CD4 epitopes that overlapped partially or completely with a predicted B cell epitope. These sequences were evaluated for their potential to induce gamma interferon production, resulting in 20 T-CD4 epitopes predicted to induce IFN-γ production. Additionally, 58 sequences were identified as potential T-CD8 epitopes showing partial or complete overlap with predicted B cell epitopes. Among these, 22 sequences were considered immunogenic T-CD8 epitopes by in silico evaluation. The complete list of predicted T-CD4 and T-CD8 epitopes and their respective information is in Supplementary Table S2.

### 2.3. Selected Sequences with B, T-CD4 and T-CD8 Epitopes

Based on the predicted B-cell, T-CD4, and T-CD8 epitopes, we selected 16 sequences in which B-cell epitopes overlapped with T-cell epitopes, defining them as vaccine candidates, including 7 epitopes from SNV, 2 of ANDV, 4 of SEOV, and 3 of PUUV, shown in Table 2. Epitope coordinates in Table 2 denote the extended vaccine window that encompasses the overlapping B- and T-cell segments and may therefore differ by a few residues from the core linear B-cell epitope coordinates listed in Table 1.

### 2.4. Evaluation of Allergenicity and Toxicity of Defined Epitopes

To ensure the safety of the proposed vaccine, each selected epitope was evaluated in silico for allergenicity, toxicity, and hemotoxicity using the AllergenFP, ToxinPred, and HemoPI tools, respectively. Based on these results, five epitopes were excluded from further consideration: SNV_(914–928)_, SNV_(1060–1070)_, SEOV_(160–172)_, and SEOV_(1045–1063)_ due to predicted allergenicity, and PUUV_(740–757)_ due to predicted toxicity (Table 3). Following this rigorous safety screening, a final set of 11 non-allergenic, non-toxic, and non-hemotoxic epitopes was selected to compose the chimeric protein designed as a universal hantavirus vaccine candidate.

### 2.5. Population Coverage

**Figure 1:**
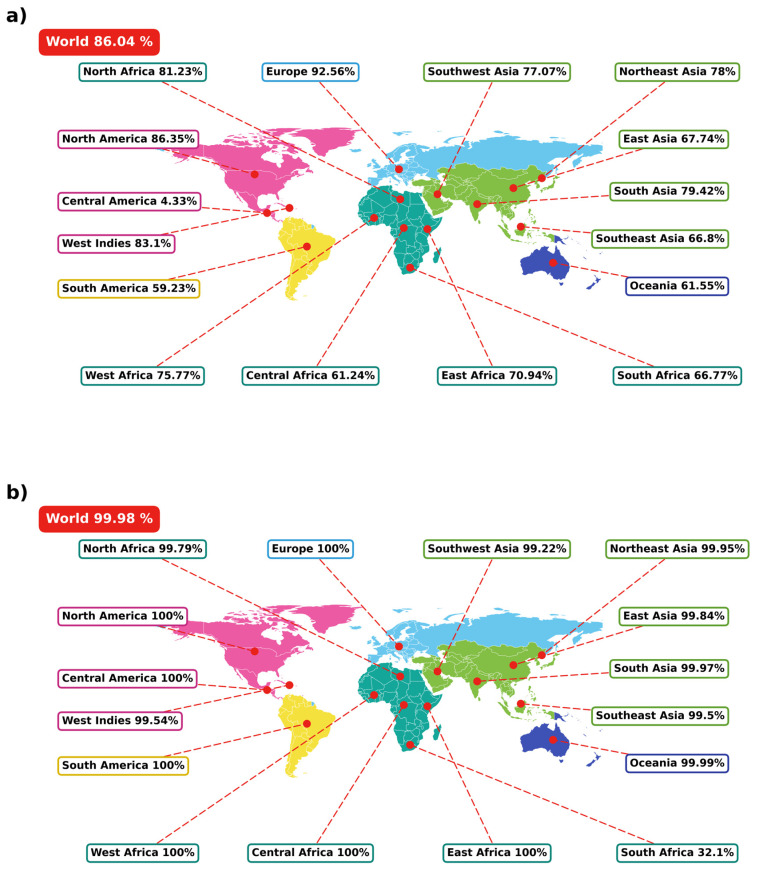
Diagrammatic representation of the coverage of the global population of selected (**a**) HLA-I and (**b**) HLA-II epitopes.

Population coverage was estimated using the IEDB analysis tool, based on the predicted binding of T-cell epitopes to HLA class I and II alleles. The results showed that the coverage in the world population presented for MHC I was 86.4% (Figure 1a), and MHC II was 99.98% (Figure 1b). Regarding the population of Europe and Asia, where HFRS is a critical problem, the coverage presented for HLA-I ranged from 66.80% to 92.56%. In comparison, the coverage presented for HLA-II ranged from 99.22% to 100%. In the Americas, where HPS is a major health concern, HLA-I coverage ranged from 4.33% in Central America to 86.35% in North America. In contrast, HLA-II coverage reached up to 100% across all American subregions. The comparatively low HLA class I coverage estimated for Central America (4.33%) should be interpreted with caution, as it largely reflects the limited HLA-A and HLA-B allele-frequency data available for this region in the IEDB reference set rather than a true absence of responders; the near-complete HLA class II coverage and the high combined coverage in the same populations mitigate this gap. The full data of Population Coverage Analysis is summarized in Supplementary Tables S3 and S4. These findings highlight the broad immunogenetic reach of the selected epitopes, particularly for MHC class II, supporting their potential utility in a broadly effective hantavirus vaccine.

### 2.6. Epitope Conservation in HPS and HFRS-Associated Hantaviruses

To investigate the potential for cross-reactivity among epitopes from hantaviruses associated with human disease, we compared the identity of selected epitopes with GPs from SNV, ANDV, LANV, CHOV, and RIOMV, associated with HPS, and DOBV, TULV, HTNV, and THAIV associated with HFRS. Each selected vaccine epitope showed at least moderate conservation with one or more hantavirus species analyzed. The five SNV and two ANDV epitopes were highly conserved across HPS-associated hantaviruses, with identity values ranging from 57% to 100%. The epitopes located in the C-terminus, SNV_(680–695)_ and ANDV_(692–700)_, were moderately to highly conserved in HFRS-associated hantaviruses (62% to 73% of identity). Regarding the epitopes predicted in HFRS hantavirus, the SEOV epitopes presented high levels of identity with HANTV, DOBV, and THAIV (ranging from 67% to 100%). In addition, the epitopes PUUV_(365–385)_ and PUUV_(940–967)_ were only highly conserved with TULAV (identities of 95% and 71%, respectively). Interestingly, PUUV-derived epitopes showed greater conservation with HPS-associated hantaviruses (46% to 67%) than with other HFRS-associated viruses (38% to 47%), suggesting potential cross-reactive capacity beyond their original disease context. The degree of conservation of each epitope is detailed in Table 4. These findings support the broad cross-protective potential of the selected epitopes, reinforcing their relevance for the design of a universal hantavirus vaccine.

### 2.7. Construction of Multi-Epitope Vaccine

**Figure 2:**
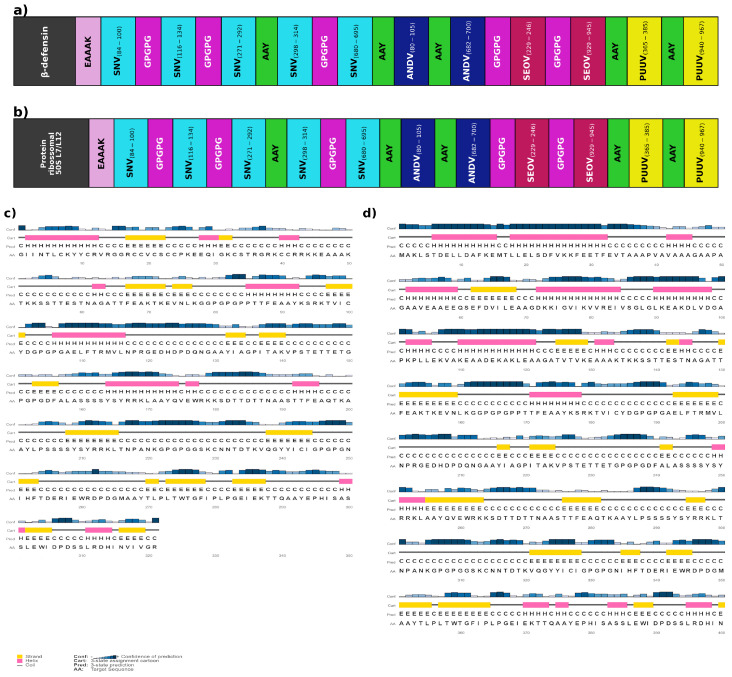
Epitope architecture and secondary-structure prediction of the multi-epitope vaccine constructs. (**a**) Linear schematic of VC1: β-defensin adjuvant fused via EAAAK to the multi-epitope backbone. (**b**) Linear schematic of VC2: 50S ribosomal protein L7/L12 adjuvant fused via EAAAK to the multi-epitope backbone. Epitopes blocks derived from SNV, ANDV, SEOV and PUUV are separated by GPGPG and AAY linkers. Blocks are color-coded by source virus and linker type as indicated: SNV (cyan blocks), ANDV (blue blocks), SEOV (Dark-red blocks), PUUV (yellow blocks), structural-adjuvant (gray block), EAAAK-linker (light pink blocks), GPGPG-linker (purple blocks), AAY-linker (green blocks). (**c**) Secondary-structure prediction for VC1 and (**d**) VC2, with per-residue confidence bars (blue) overlaid on predicted α-helices (magenta), β-strands (yellow) and coil (grey).

The basic structure of the vaccine was defined by combining the 11 selected epitopes—SNV_(84–111)_, SNV_(116–134)_, SNV_(271–292)_, SNV_(298–314)_, SNV_(680–695)_, ANDV_(80–105)_, ANDV_(682–700)_, SEOV_(229–246)_, SEOV_(929–945)_, PUUV_(365–385)_, PUUV_(940–967)_—with a suitable linker, such as GPGPG and AAY, by providing enough space and flexibility between the peptides to fold correctly, resulting in a basic sequence of 271 amino acid residues. The GPGPG linker was used to connect T-helper and B-cell epitopes, enhancing flexibility and antigen presentation, while AAY was used between TCD8 epitopes to facilitate proteasomal processing and epitope release [19]. In addition, an adjuvant molecule was attached to the C-terminal domain of the multi-epitope vaccine through an EAAAK linker to increase its potential to induce a good immune response. The vaccine construct 1 (VC1) utilizing the β-defensin adjuvant was 321 amino acids in length (Figure 2a), whereas vaccine construct 2 (VC2) utilizing the 50Srp consisted of 405 amino acids (Figure 2b). Structurally, the secondary structure prediction indicated that VC1 comprises 19.31% α-helix, 22.43% β-strand, and 58.26% coil (Figure 2c), whereas VC2 comprises 26.42% α-helix, 22.72% β-strand, and 50.86% coil (Figure 2d).

**Figure 3:**
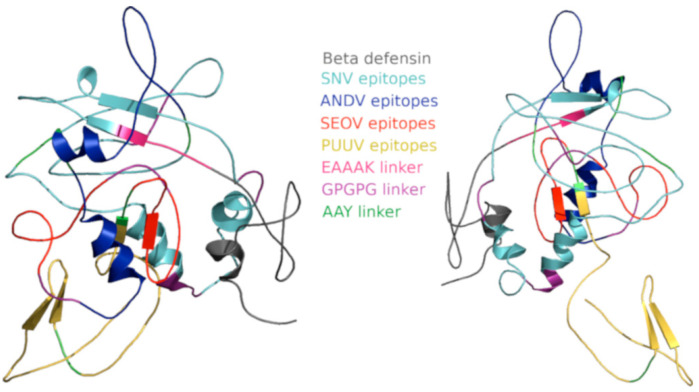
Three-dimensional architecture of the β-defensin multi-epitope construct (VC1). Cartoon representation of VC1 colored as in the linear maps: SNV (cyan), ANDV (dark blue), SEOV (red), PUUV (yellow); linkers GPGPG (purple), AAY (green), EAAAK (light rose); β-defensin adjuvant (gray). The right panel corresponds to a 180° rotation of the left panel, shown to expose complementary aspects of topology and epitope layout.

**Figure 4:**
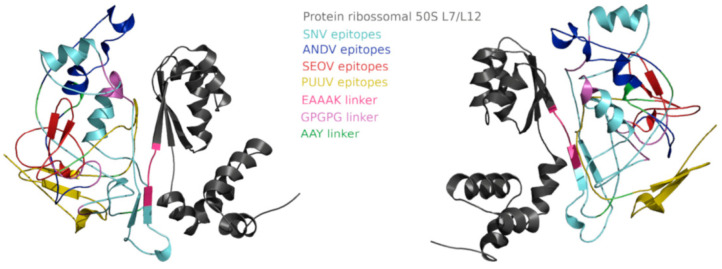
Three-dimensional architecture of the 50S ribosomal protein multi-epitope construct (VC2). Cartoon representation of VC2 colored as in the linear maps: SNV (cyan), ANDV (dark blue), SEOV (red), PUUV (yellow); linkers GPGPG (purple), AAY (green), EAAAK (light rose); 50S ribosomal protein (gray). The right panel corresponds to a 180° rotation of the left panel, shown to expose complementary aspects of topology and epitope layout.

The modelled three-dimensional structures of VC1 and VC2 are shown in Figure 3 and Figure 4, respectively. Independent quality checks indicated acceptable global geometry with local imperfections concentrated in flexible/linker segments. Ramachandran analysis showed that 99.1% (VC1) and 98.3% (VC2) of residues fall within allowed regions (Supplementary Figures S1 and S2). QMEAN4 Z-scores were −4.47 for VC1 and −2.68 for VC2, consistent with medium-quality models of chimeric, linker-rich constructs. In addition, ProSA Z-scores were −5.13 (VC1) and −5.90 (VC2); ERRAT scores were 76.67 (VC1) and 83.38 (VC2); and Verify3D compatibility was 73% (VC1) and 76% (VC2), highlighting localized environment mismatches mainly in flexible regions.

### 2.8. Physicochemical Properties

To determine the safety and efficacy of a vaccine candidate, it is crucial to evaluate various physicochemical properties. In this study, ExPASy’s ProtParam (web version, accessed on 4 August 2026; ExPASy, SIB Swiss Institute of Bioinformatics, Lausanne, Switzerland) was used to predict the physicochemical parameters of the vaccine constructs, and the results are summarized in Table 5. The multi-epitope base (MEB) used to design the vaccine constructs had a molecular weight of 29,112 Da, while the molecular weights of VC1 and VC2 were 34,712.91 Da and 42,934.09 Da, respectively. All chimeric antigens were predicted to be stable and hydrophilic, as indicated by instability indexes of less than 40 and negative GRAVY values. Moreover, all constructs were predicted to be non-allergenic and antigenic, with antigenicity scores above the reference threshold (>0.4), supporting their potential as safe and immunogenic vaccine candidates. Regarding the estimated half-life in mammalian reticulocytes, VC1 and VC2 presented a longer half-life (30 h) than the multi-epitope base (7.2 h). Notably, VC2 exhibited the highest aliphatic index (69.78), suggesting greater thermostability. In contrast, MEB (53.76) and VC1 (52.99) had lower indices, indicating reduced thermal stability, yet still within acceptable ranges for recombinant vaccines.

### 2.9. Molecular Docking and Interface Metrics

**Figure 5:**
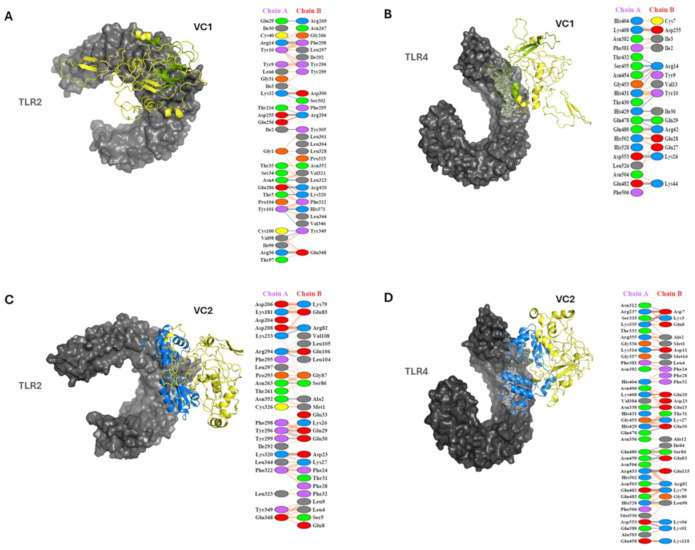
Molecular Docking of vaccine constructs (VC1 and VC2) with TLR2 and TLR4. (**A**) shows the binding mode of VC1 to TLR2, (**B**) shows the binding mode of VC1 to TLR4, (**C**) shows the binding mode of VC2 to TLR2, and (**D**) shows the binding mode of VC2 to TLR4. The structures in figures (**A**,**B**) show VC1 in yellow, with the β-defensin adjuvant shown in green, bound to TLR2 and TLR4, which are the larger gray structures shown in a-b, respectively. In the structures shown in figures (**C**,**D**), VC2 is represented in yellow, with the adjuvant 50Srp in blue, bound to TLR2 and TLR4, which are the larger structures in gray shown in c–d, respectively.

ClusPro 2.0 produced well-populated clusters (members: 68–173) for all complexes with favorable weighted scores. Meanwhile, PRODIGY web server estimated binding affinity values (ΔG) between −12.3 and −15.6 kcal/mol and dissociation constants (Kd) between 2.2 × 10^−9^ and 9.3 × 10^−12^ M at 37 °C. These results support compatible vaccine–TLR engagements (Table 6). CoCoMaps2 was used to quantify interfaces and indicated a buried surface area (BSA) upon complex formation between 1888.8 and 3162.6 Å^2^ and an interface area between 944.4 and 1581.3 Å^2^ across the four complexes. The total number of interacting residues was between 53 and 84, with 33 to 47 residues on the TLR and 20 to 37 residues on the vaccine construct. VC2–TLR4 exhibited the largest BSA (3162.6 Å^2^) and the highest number of interacting residues (84). VC1–TLR2 and VC2–TLR2 also presented extensive interfaces (BSA: 2962.4 and 2854.8 Å^2^, respectively). The polar fraction of the interface was within the expected range for ectodomain recognition (35–45%; with the remainder non-polar). In addition, the contact counts were consistent with the stabilizing networks observed in protein–TLR complexes. The geometry and orientation of the docked poses, as well as the per-residue contact maps (PDBsum), are shown in Figure 5. In addition, CoCoMaps 2.0 provided an atomic-level interaction landscape for each docked interface. Across complexes, the interfaces were stabilized by a combination of polar and non-polar contacts, including 8–13 hydrogen bonds, 3–9 salt bridges, and 7–20 apolar van der Waals contacts, together with 1–3 π–π and 1–2 CH–π interactions. The number of proximal contacts ranged from 36 to 68, and clashes were low overall (1–7), indicating limited steric conflicts in the selected poses. Notably, VC2–TLR4/MD-2 exhibited the richest polar contact network (13 H-bonds; 9 salt bridges; 22 CH–O/N bonds) alongside a large interface (BSA 3162.6 Å^2^), consistent with a well-stabilized docked arrangement. The results obtained from the ClusPro 2.0, PRODIGY web server, and CoCoMaps 2.0 servers are summarized in Table 6, and the complete CoCoMaps2 contact report is provided in Supplementary Table S5.

### 2.10. Analysis of Vaccine Constructs Using C-Immun Simulator

In our study, we used the C-ImmSim 10.1 simulator to evaluate the immunogenic efficacy of two vaccine constructs: VC1 and VC2. The vaccines were administered in three doses on days 0, 30, and 60. This tool allowed us to predict key immune parameters, including T-cell activation and proliferation, as well as antibody production. This enabled us to make a comparative assessment of both constructs.

**Figure 6:**
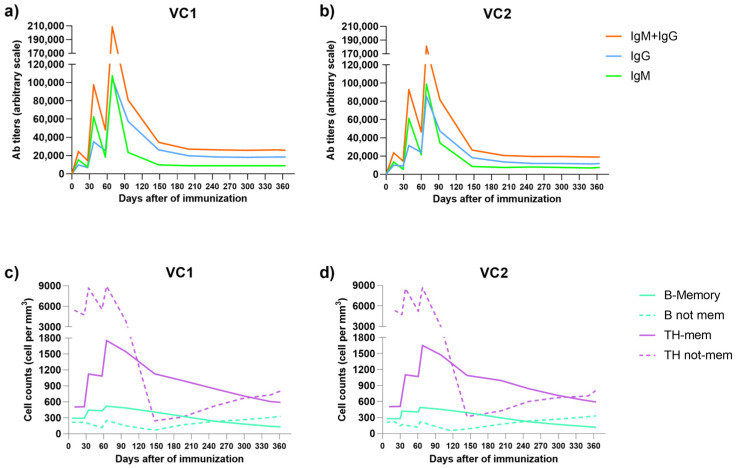
In silico simulation of immune responses induced by the vaccine constructs VC1 and VC2. Graphs were generated by the C-ImmSim server following three simulated administrations of the chimeric vaccines on days 0, 30, and 60. Panels (**a**,**b**) show the dynamics of antibody titers (IgM, IgG sub-classes, and combined immunoglobulin concentrations) for VC1 and VC2, respectively. Panels (**c**,**d**) represent the kinetics of T helper (CD4+) cell population expansion for VC1 and VC2, respectively.

Figure 6 summarizes the main immune response profiles elicited by VC1 (panels a and c) and VC2 (panels b and d). In silico, both constructs triggered a progressive increase in T helper cell responses following each immunization, with peak activation occurring around day 70 (Figure 6c,d). Similarly, antibody titers rose cumulatively, with each subsequent dose resulting in a higher peak. This ultimately generated a strong, sustained humoral response that remained elevated beyond day 90.

Both vaccine constructs were capable of eliciting robust cellular and humoral immune responses. VC2 induced faster and more pronounced early T-cell activation, particularly after the first and second doses. In contrast, VC1 promoted a more persistent antibody response over time. These findings suggest that VC2 may provide rapid immune activation, while VC1 may confer more durable protection. These differences highlight the critical role of adjuvant selection in determining the magnitude and duration of vaccine-induced immunity.

No other significant discrepancies were observed between the immune simulations of VC1 and VC2. A comprehensive overview of the simulated immune parameters is provided in Supplementary Figures S3 and S4.

## 3. Discussion

In recent years, RNA viruses have rapidly emerged and disseminated, posing a significant threat to human health [20]. Of particular concern are the hantaviruses, which are responsible for the causation of both pulmonary syndrome (HPS) and hemorrhagic fever with renal syndrome (HFRS) [21]. These syndromes are associated with different hantavirus species, but both can be lethal in up to 50% of cases [7,20,21]. Although inactivated hantavirus vaccines have been licensed for human use in China and Korea, a widely available vaccine for hantavirus infection remains unavailable, despite extensive vaccine research [20,22]. In this scenario, multi-epitope vaccines offer a promising solution to the limitations of traditional vaccines, as they enable the combination of epitopes from different viruses or antigens, enhancing cross-reactivity and broadening protective immune responses. Additionally, multi-epitope vaccines provide an effective strategy to overcome challenges related to in vitro antigen expression and the complexity of pathogen cultivation [12,13]. Recent studies employing this approach have yielded promising results regarding vaccine efficacy [23,24,25].

Using computational tools, this study designed a multi-epitope vaccine capable of eliciting both humoral and cellular immune responses against hantaviruses responsible for the two major clinical forms, HPS and HFRS. In recent years, several multi-epitope vaccine candidates against hantaviruses have been proposed; however, most of these efforts have focused on a single hantavirus species [26,27,28,29,30]. The protective immune response against a single hantavirus is typically limited to closely related species, due to the substantial genetic variability in viral proteins between the causative agents of HPS and HFRS [1,31,32]. In this context, whereas previous studies have typically focused on combining epitopes from the nucleoprotein and glycoprotein (GP) of a single hantavirus species, our study targeted a combination of epitopes derived exclusively from the GPs of multiple hantavirus species. This protein plays a critical role in viral entry into host cells and is considered a key target for novel therapies and vaccine development against hantavirus infection [1,7]. However, it is poorly conserved among hantaviruses, which contributes to differences in infectivity between species associated with HPS and HFRS and poses a challenge for vaccine development [1,31,32]. To develop a vaccine capable of providing protection against both HPS and HFRS, we predicted epitopes from the glycoproteins (GPs) of four hantavirus species: SNV and ANDV, the primary etiological agents of HPS in North and South America, respectively [33]; SEOV, which causes HFRS in the Old World [34]; and PUUV, associated with nephropathia epidemica, a milder HFRS-like illness prevalent throughout Europe [17]. Based on these proteins, we selected 11 vaccine epitopes that combine B- and T-cell epitopes and are conserved among other hantaviruses associated with human disease. The inclusion of both B- and T-cell epitopes within selected sequences has been employed in previous multi-epitope vaccine designs, including those targeting hantaviruses [35]. Similarly, the combination of epitopes from different hantavirus species has also been explored, although limited to viruses causing HFRS [36]. To the best of our knowledge, the vaccine proposed in this study is the first to aim for cross-protection against both HFRS and HPS. Unlike earlier hantavirus multi-epitope designs, which have been restricted to a single species or to viruses causing only HFRS and have typically combined nucleoprotein and glycoprotein determinants, the present construct is assembled exclusively from glycoprotein epitopes drawn from both New World (SNV, ANDV) and Old World (SEOV, PUUV) species and is explicitly selected for cross-conservation across HPS- and HFRS-associated hantaviruses; this dual-syndrome, glycoprotein-focused strategy constitutes the specific novelty of our approach.

Regarding the epitope prediction methods, our group has successfully identified and validated epitopes in viruses [37,38,39], bacteria [40,41], and protozoa [42,43,44]. Based on this expertise, we defined 11 sequences as vaccine epitopes capable of eliciting cross-protective responses against the major hantaviruses associated with HPS and HFRS. Notably, some of the SEOV and PUUV epitopes predicted in our study partially or fully matched sequences reported in previous Old World hantavirus vaccine studies [26,27,28], supporting the accuracy of our predictions. Similarly, residues within SNV and ANDV epitopes identified here have been previously described as targets of neutralizing antibodies against New World hantaviruses [45]. Moreover, the strategy used for TCD4 and TCD8 epitope prediction has been employed in other studies, reinforcing our epitope selection and its potential to provide broad global coverage [46,47].

Several methodological choices were made deliberately to strengthen epitope selection. Linear B-cell epitopes were retained only when predicted by at least three of six independent algorithms, a consensus strategy that reduces the method-specific false-positive rate of any single predictor, and were further filtered with VaxiJen at the 0.4 threshold recommended for viral antigens. For T-cell epitopes, TepiTool percentile-rank cut-offs (percentile = 10 for MHC class II and = 1 for class I) and a requirement of recognition by at least nine frequent HLA alleles were applied to favor broadly presented peptides, while IFN-gamma and class I immunogenicity filters prioritized functionally relevant determinants. Robetta and GalaxyRefine were chosen for modelling and refinement given their consistent performance in CASP assessments, ClusPro for its validated PIPER-based sampling, and C-ImmSim for agent-based immune simulation; each is a widely benchmarked tool in its category, which facilitates comparison with previous multi-epitope studies.

Given the diversity of HLA alleles and their distribution across different ethnic populations, population coverage analysis is essential in multi-epitope vaccine design [48]. Recently, Ismail et al. proposed a hantavirus vaccine with 98.55% global coverage [47]; however, that study assessed only the combined coverage of TCD4 and TCD8 epitopes. In contrast, we selected 11 epitopes combining both MHC class I and II epitopes for this analysis. Our results demonstrated 100% combined global (HLA class I plus class II) population coverage, and we additionally evaluated coverage separately for TCD4 and TCD8 epitopes. The combined value exceeds the coverage of either class alone because an individual is counted as covered when responding through HLA class I or class II; the union of both estimates therefore surpasses each separate value shown in Figure 1. These findings support the broad-spectrum potential of our vaccine candidate and its applicability across ethnically diverse populations. Importantly, this is the first study to explore epitope conservation across hantaviruses in the context of both HPS and HFRS, reinforcing the rationale for a cross-protective, multi-epitope vaccine.

After defining the vaccine epitopes, we designed the multi-epitope constructs. To ensure proper folding and structural flexibility, the selected epitopes were joined using specific linkers: GPGPG and AAY. TCD8 epitopes were linked using the AAY linker, while B-cell and TCD4 epitopes were fused using GPGPG, as described in previous studies [47,49]. The AAY linker facilitates the natural formation of epitopes and minimizes the generation of junctional epitopes, thereby improving antigen presentation. GPGPG, a glycine-rich linker, increases solubility and provides structural flexibility and accessibility to adjacent domains [50].

The epitopes were arranged in blocks grouped by source virus and epitope class, with cytotoxic determinants joined by AAY and helper/B-cell determinants by GPGPG, so that immunodominant and highly conserved segments were distributed along the construct rather than clustered. Because the GPGPG and AAY linkers are specifically intended to prevent the formation of neo-junctional epitopes and to promote independent folding and proteasomal processing of each determinant, the linear order of the epitopes is expected to have a limited effect on the antigenic content of the construct: reordering would preserve the same set of B- and T-cell determinants and, given the flexible linkers, is unlikely to change the predicted immunogenicity, although it could locally influence folding and is therefore best assessed experimentally.

In addition, we in silico fused the multi-epitope sequence to a selected adjuvant via the rigid EAAAK linker, generating two computationally designed constructs. To identify the optimal vaccine construct, we evaluated two adjuvants: (1) β-defensin, known to activate innate antiviral responses and enhance adaptive immunity [51], and (2) 50S ribosomal protein L7/L12 (50Srp), which has been reported to increase the immunogenicity of multi-epitope vaccines [52]. Both vaccine constructs—VC1 (with β-defensin) and VC2 (with 50Srp)—were predicted to be stable and soluble. However, VC2 showed higher thermostability compared to VC1, a pattern also reported by Ismail et al. when comparing these same adjuvants [47]. It is worth noting that aliphatic index values between 50 and 150, as observed for VC1 and VC2, are considered favorable [53] and have been consistently reported in other multi-epitope vaccine designs [54,55,56].

Given the predicted physicochemical properties of both constructs, we further explored in silico the potential contribution of each selected adjuvant to immunogenicity. We first assessed the potential binding of VC1 and VC2 to Toll-like receptors (TLRs) 2 and 4. TLR agonists are commonly used as adjuvants in vaccines to enhance the magnitude and quality of the immune response [57,58]. TLR2 and TLR4 are host receptors involved in immune modulation through the recognition of pathogen-associated molecular patterns (PAMPs) derived from microbial cells [59]. In this study, molecular docking analysis demonstrated that both constructs exhibited strong binding affinity and favorable interactions with these receptors, with VC1 showing superior interaction with TLR2 compared to VC2. Additionally, normal mode analysis revealed that both VC1 and VC2 possess sufficient structural flexibility to interact effectively with the host immune environment.

To further evaluate the immunogenic potential of the vaccine constructs, we performed immune simulations using the C-ImmSim 10.1. Simulated immunizations consisted of one initial dose followed by two boosters on days 30 and 60, with immune responses monitored for one year. The simulation predicted robust and long-lasting antibody responses and immune cell activation induced by both constructs. Notably, VC1 elicited a higher and more sustained antibody response over time, suggesting its potential to induce longer-lasting protection. Both VC1 and VC2 demonstrated comparable capacities to stimulate T cell differentiation, proliferation, and cytokine production. This strategy has been widely adopted in the development of multi-epitope vaccines, and our findings are consistent with results reported in similar studies [53,56,60].

Although the pipelines cited above are largely computational, immunoinformatics-guided multi-epitope constructs designed with comparable workflows have progressed to experimental testing; for example, recombinant multi-epitope proteins have elicited protective, antigen-specific responses in animal models of foot-and-mouth disease virus and Helicobacter pylori infection [24,25]. In addition, our group has experimentally identified and validated naturally immunogenic B-cell epitopes in hantaviruses and in other viral, bacterial and protozoan pathogens [37,38,39,40,41,42,43,44], which supports the translational plausibility of the present predictions. We nonetheless emphasize that the constructs proposed here remain to be produced and tested in vitro and in vivo before any protective claim can be made.

Regarding the limitations of our study, we deliberately did not use normal-mode analysis (NMA) in this work—unlike some in silico studies—because NMA, when applied directly to docked static complexes, can be misleading and is more appropriate as an interpretive add-on to replicated molecular dynamics (MD) trajectories. A key limitation, therefore, is the absence of MD simulations in this pre-experimental stage. More broadly, the predictions reported here carry intrinsic uncertainty: epitope, antigenicity, allergenicity and immunogenicity predictors have finite sensitivity and specificity and are sensitive to the chosen score thresholds; population-coverage estimates depend on the completeness of HLA allele-frequency reference data; and the docking and C-ImmSim analyses rely on simplified scoring functions and agent-based assumptions. These outputs should therefore be regarded as hypothesis-generating and prioritized for experimental confirmation rather than as definitive measures of protective efficacy. Even so, the recognized adjuvant potential of the selected structural modules (β-defensin or L7/L12) and the favorable physicochemical profiles of the multi-epitope constructs support their advancement to animal testing as a hypothesis-driven next step. We also note that our design explicitly targets broad protection across Old- and New-World hantaviruses, which raises the bar for experimental validation. Accordingly, wet-lab studies (peptide/protein ELISAs, neutralization, and T-cell functional assays, followed by in vivo evaluation) are required to confirm and refine the in silico predictions presented here.

In summary, the in silico-designed vaccine candidates proposed here represent a promising starting point toward broad-spectrum hantavirus vaccines; however, all conclusions are computational and require rigorous in vitro/in vivo validation before any claims about protective efficacy can be made. Nonetheless, the observed differences in thermostability and antibody induction between the constructs underscore the need for in-depth experimental validation. We believe that the computational pipeline presented in this study can be further leveraged to develop novel multi-epitope vaccines targeting other neglected pathogens.

## 4. Materials and Methods

### 4.1. Studied Proteins

The sequences of the GPs of ANDV (GenBank ID: WNV26843.1), SNV (GenBank ID: AIA08879.1), SEOV (GenBank ID: UXK58064.1), and PUUV (GenBank ID: ALJ30176.1) were downloaded from the GenBank database (www.ncbi.nlm.nih.gov/genbank/ (accessed on 15 May 2023)), and the exposed regions of these proteins were used to predict epitopes for B cells and T cells in the next phase of our study.

### 4.2. B Cell Epitope Prediction

Linear B cell epitopes were predicted using a combination of six algorithms: ABCpred (http://crdd.osdd.net/raghava/abcpred/ (accessed on 16 May 2023) [61], Bepipred (Bepi 1.0) [62] and Emini Surface Accessibility prediction (ESA) [63], from the Immune Epitope Database (IEDB) (http://tools.iedb.org/bcell/ (accessed on 16 May 2023), Bepipred 3.0 (Bepi 3.0, https://services.healthtech.dtu.dk/services/BepiPred-3.0/ (accessed on 16 May 2023) [64], LBTOPE [65] (https://webs.iiitd.edu.in/raghava/lbtope/index.php (accessed on 17 May 2023), and SVMTrip [66] (http://sysbio.unl.edu/SVMTriP/index.php (accessed on 17 May 2023). All predictions were performed using the default parameters provided by each respective tool. In addition, antigenicity of the predicted peptides was assessed using VaxiJen (http://www.ddg-pharmfac.net/vaxijen/VaxiJen/VaxiJen.html (accessed on 25 May 2023), an alignment-independent tool based on physicochemical properties of proteins, with a threshold set at 0.4 [67].

Only sequences predicted as epitopes by at least three of the six algorithms and classified as antigenic (VaxiJen score ≥ 0.4) were retained. From these, we selected peptides with a minimum length of 9 amino acids to ensure immunogenic potential.

### 4.3. T-CD4 Epitope Prediction

The activation of T helper cells (T-CD4) is contingent upon their recognition of a peptide bound to an HLA class II molecule. These peptides are derived from extracellular proteins and are designed as T-CD4 epitopes [68]. In this study, we employed the online computational MHC binding prediction tool, TepiTool, from IEDB (http://tools.iedb.org/tepitool/ (accessed on 10 June 2024) [69] to predict T-CD4 epitopes in the studied hantavirus GP. In summary, the full-length sequence of each studied GP was submitted to TepiTool along with a panel of the 26 most frequent HLA class II alleles in the global population. This panel corresponds to the alleles DR (DRB1*01:01, DRB1*03:01, DRB1*04:01, DRB1*04:05, DRB1*07:01, DRB1*08:02, DRB1*09:01, DRB1*11:01, DRB1*12:01, DRB1*13:02, DRB1*15:01, DRB3*01:01, DRB3*02:02, DRB4*01:01, DRB5*01:0), DP (DPA1*01/DPB1*04:01, DPA1*01:03/DPB1*02:01, DPA1*02:01/DPB1*01:01, DPA1*02:01/DPB1*05:01, DPA1*03:01/DPB1*04:02) and DQ (DQA1*01:01/DQB1*05:01, DQA1*01:02/DQB1*06:02, DQA1*03:01/DQB1*03:02, DQA1*04:01/DQB1*04:02, DQA1*05:01/DQB1*02:01, DQA1*05:01/DQB1*03:01). Peptides were predicted based on 15-mer sliding windows, and only those with a percentile rank ≤ 10 were retained, indicating high binding affinity to HLA class II molecules [69]. The selected peptides were further evaluated for their capacity to induce IFN-γ responses using the IFNepitope server (https://webs.iiitd.edu.in/raghava/ifnepitope/application.php, accessed on 16 June 2024).

To define high-confidence vaccine candidates, we selected only those T-CD4 epitopes that met all three criteria simultaneously: (i) recognition by at least nine distinct HLA class II alleles, (ii) positive prediction for IFN-γ induction, and (iii) spatial proximity or partial overlap with predicted B cell epitopes. This integrative filtering strategy ensured the inclusion of T-CD4 epitopes with broad population coverage, robust immunogenicity, and potential to support coordinated B cell responses.

### 4.4. T-CD8 Epitope Prediction

The activation of cytotoxic T lymphocytes (T-CD8) depends on the recognition of antigenic peptides presented by HLA class I molecules. These peptides are typically 8–11 amino acids long and are derived from intracellular proteins processed via the proteasomal pathway [68]. In this study, T-CD8 epitopes were predicted using the MHC class I binding tool (TepiTool) from IEDB, applying the complete amino acid sequences of the glycoproteins and a reference panel of the 27 most frequent HLA class I alleles globally. These alleles—covering loci HLA-A and HLA-B—represent the main MHC class I supertypes: A01:01, A02:01, A02:03, A02:06, A03:01, A11:01, A23:01, A24:02, A26:01, A30:01, A30:02, A31:01, A32:01, A33:01, A68:01, A68:02, B07:02, B08:01, B15:01, B35:01, B40:01, B44:02, B44:03, B51:01, B53:01, B57:01, and B*58:01. Epitope candidates were selected based on a predicted binding percentile rank ≤ 1.0, indicating high affinity to MHC class I molecules.

To further refine the selection, predicted epitopes were evaluated using the IEDB Class I Immunogenicity tool, which estimates the likelihood of an epitope to elicit a T cell response based on amino acid features at key anchor positions. Only epitopes with positive immunogenicity scores were retained. Additionally, spatial proximity to predicted B cell epitopes was considered, based on the rationale that overlapping or adjacent epitopes may enhance helper and cytotoxic T cell responses when presented in a multi-epitope vaccine construct.

### 4.5. Definition of Vaccine Epitopes

Given the location of predicted B-cell and T-cell epitopes, we evaluated the overlap of amino acid residues of these epitopes. Vaccine epitopes were defined as regions of B-cell epitopes that overlapped partially or completely with predicted T-CD4 or T-CD8 epitopes, thereby maximizing the likelihood of inducing a coordinated humoral and cellular immune response. This overlap-based criterion is grounded in the principle of linked recognition: because B cells internalize antigens through their surface immunoglobulin and present derived peptides on MHC class II to cognate T-helper cells, positioning a T-helper (and, where possible, a cytotoxic) determinant within or adjacent to a B-cell epitope favors intramolecular help, promoting class switching, affinity maturation and durable antibody responses while also priming coordinated CD8+ T-cell responses.

### 4.6. Evaluation of Allergenicity, Toxicity and Hemotoxicity

Vaccine epitopes sequences were in silico evaluated for allergenicity, toxicity, and hemotoxicity, using the AllergenFP algorithm (https://ddg-pharmfac.net/AllergenFP/ (accessed on 2 July 2024), ToxinPred server (https://webs.iiitd.edu.in/raghava/toxinpred/algo.php (accessed on 2 July 2024) and the HemoPi server (https://webs.iiitd.edu.in/raghava/hemopi/ (accessed on 2 July 2024), respectively [70,71,72]. Sequences predicted to be allergenic, toxic, or hemotoxic were excluded from the vaccine design.

### 4.7. Conservation Analysis

Conservancy is defined as the fraction of protein sequences that contain the epitope, and Identity is the degree of correspondence between two sequences [46]. The IEDB epitope conservation analysis tool (http://tools.iedb.org/conservancy/ (accessed on 8 July 2024) was used to evaluate the identities of predicted sequences in comparison with the GP sequences of other hantavirus species related to diseases in humans, obtained from the GenBank database. The identities of selected epitopes were compared to the GP sequences of *O. chocloense* (CHOV) (Genbank ID: APD78411.1) [73] and *O. mamorense* (RIOMV) (Genbank ID: ACU46022.1) [74] related to HPS, and *O. dobravaense* (DOBV) (Genbank ID: ADP21265.1) [75], *Orthohantavirus tulaense* (TULV) (Genbank ID: NP_942586.1) [76,76], *Orthohantavirus hantanense* (HTNV) (Genbank ID: P28728) [77,78], and *Orthohantavirus thailandense* (THAIV) (GenBank ID: QZA58117) [79,80] related to HFRS.

### 4.8. Population Coverage Analysis

Developing vaccines that target T-cell epitopes requires a careful selection of multiple epitopes with varied HLA-binding specificities. This approach ensures broader coverage of the patient population, as HLA molecules are highly polymorphic and specific HLA alleles are expressed at different frequencies across various ethnicities [48]. For the vaccination coverage analysis of the epitopes predicted in this study, we made use of the IEDB Analysis for Population Coverage (http://tools.iedb.org/population/ (accessed on 10 July 2024) feature. This tool calculates the percentage of individuals likely to respond to a given set of epitopes based on HLA genotypic frequencies. The program evaluates coverage in three modes (Separate Class I, Separate Class II, and Class I and II combined), and calculates the projected population coverage, the mean number of occurrences of HLA epitopes/combinations recognized by the population, and the minimum number of occurrences of HLA epitopes/combinations recognized in 90% of the population [48]. Here, we investigated the population coverage of each selected T cell epitope (T-CD4 and T-CD8 epitopes) based on HLA alleles predicted as recognizer to each epitope.

### 4.9. Designing of Multi-Epitope Vaccine

To create the final vaccine sequence, we used the predicted B-cell epitopes as a template for CTL and HTL epitopes. We shortlisted and selected the epitopes whose sequences overlapped with the B-cell epitopes. These selected epitopes were used in the final vaccine construction [81]. In our study, we constructed the vaccine by joining the selected CTL, HTL, and B-cell epitopes using AAY and GPGPG linkers. The AAY linker joined the T-CD8 epitopes, while the GPGPG linker joined T-CD4 and B-cell T-cell epitopes, respectively [35]. Additionally, to effectively activate both innate and adaptive immune responses, a strong immunostimulant adjuvant should be added to enhance immunogenicity [47]. For our study, we designed two vaccine constructs. Construct 1 uses the adjuvant β-defensin [51], while construct 2 uses the 50S ribosomal protein L7/L12 (50Srp) [82]. The EAAAK linker was utilized for attaching the adjuvants to the N-terminal of the vaccine, which enhances its immunogenicity. These linkers were used for construction because they facilitate epitope display, allowing effective immune processes. Moreover, these linkers also help to keep the epitopes separated and prevent them from folding [19,83,84].

### 4.10. Secondary and Tertiary Structure Prediction

The PSIPRED server [85] (http://bioinf.cs.ucl.ac.uk/psipred/ (accessed on 3 August 2024) was used to predict the secondary structure of the vaccine models, while the Robetta server [86] (https://robetta.bakerlab.org/) was used for tertiary structure modeling. Robetta prioritizes comparative modeling based on template recognition via PSI-BLAST, FFAS03, or 3D-Jury. When no suitable template is available, it switches to a de novo strategy using Rosetta fragment insertion [19,51,82,83,84,87,88]. The 3D structure was improved using the GalaxyRefine server [89] (https://galaxy.seoklab.org/cgi-bin/submit.cgi?type=REFINE (accessed on 10 August 2024). GalaxyRefine is a web-based tool that is particularly effective in refining the quality of local structures in protein models. This has been demonstrated through tests conducted on CASP refinement category targets and CASP10 server models. On average, it shows moderate improvement in the quality of the backbone structure. The server may be used to refine model structures obtained from available structure prediction methods, including the best current template-based modeling servers [89]. Global model quality was evaluated with QMEAN4 (Swiss-Model/ProQ suite), Molprobity (Ramachandran), ProSA-web (Z-score and residue-wise energy), ERRAT, and Verify3D.

### 4.11. Physicochemical and Immunological Properties

To analyze the physicochemical properties of the vaccine constructs, we used Protparam from the ExPASy server (http://web.expasy.org/protparam/ (accessed on 16 August 2024) [87]. The parameters computed by ProtParam include molecular weight, theoretical pI, amino acid composition, atomic composition, extinction coefficient, estimated half-life, instability index, aliphatic index, and the grand average of hydropathicity (GRAVY) [19]. We evaluated immunological properties such as antigenicity and allergenicity using Vaxijen [64] (http://www.ddg-pharmfac.net/vaxijen/VaxiJen/VaxiJen.html (accessed on 17 August 2024) and AllerTop 2.0 [88] (https://www.ddg-pharmfac.net/AllerTOP/ (accessed on 17 August 2024), respectively.

### 4.12. Molecular Docking of Vaccines and Toll-like Receptors

Analysis of the ability of the multi-epitope vaccine construct to bind to the antigen recognition receptors was performed using the ClusPro server (https://cluspro.bu.edu). The ClusPro algorithm computes the docking of two protein structures and provides a result as a ranked list of putative complexes. The docking is based on two main steps—prediction of ligand conformation, position and orientation, and evaluation of binding affinity. After filtering this set of structures, the complexes with favorable electrostatic and desolvation-free energies are selected [90]. To evaluate the interaction between the vaccine construct and host receptors, we selected TLR2 (TLR2/1 or TLR2/6) and TLR4/MD-2 as surface PRRs compatible with protein subunit formulations. TLR3 (endosomal dsRNA) was not targeted as it is more appropriate for nucleic-acid adjuvants. The adjuvants β-defensin (TLR2/TLR4) and L7/L12 (TLR4-biased) were chosen based on prior multiepitope vaccines studies reporting adjuvanticity via these receptors [91,92].

Docking was conducted as blind/exploratory screening, with the goal of assessing compatibility rather than identifying a native binding site. The tertiary structure of human TLR2 and TLR4 were obtained from the Research Collaboratory for Structural Bioinformatics (RCSB) Protein Data Bank (PDB: 6NIG, and PDB:3FXI, respectively), followed by the removal of the non-TLR molecules. TLRs act as antigen-recognition receptors. The ClusPro server samples billions of conformations using PIPER [93] and performs cluster analysis of the 1000 structures with the lowest energy. The largest clusters represent the most likely models of the complex; the selected structures are further refined using energy minimization. In addition, the PRODIGY server was employed to predict the binding free energy (ΔG) and dissociation constant (Kd). For each docked complex (VC1–TLR2, VC1–TLR4/MD-2, VC2–TLR2 and VC2–TLR4/MD-2), interfaces were characterized with CoCoMaps2 (https://aocdweb.com/BioTools/cocomaps2/ (accessed on 9 February 2026) [94]. The server reports the buried surface area upon complex formation (BSA) and interface area (Å^2^), the number of interacting residues on each partner, and non-covalent counts. Visualization and analysis of docking results were performed using PyMol software and PDBsum (https://www.ebi.ac.uk/thornton-srv/databases/pdbsum/ (accessed on 3 September 2024) [83,95].

### 4.13. Immune Simulation

The immunogenicity and immune response profile of a chimeric peptide vaccine was characterized using the C-ImmSim service (https://kraken.iac.rm.cnr.it/C-IMMSIM/index.php (accessed on 13 September 2024). The C-ImmSim server is an in silico-based immune simulation method [96,97]. At three different intervals for one year, three injections of a VC were administered on days 1, 30, and 60, keeping all simulation parameters at default without LPS, volume, and simulation stages at 10 and 1000, respectively, with homozygous host haplotypes HLA-A*03:01, HLA-A*33:01, HLA-B*35:01, HLA-B*53:01, HLA-DRB1*04:01, and HLA-DRB5*01:01 [98].

## Figures and Tables

**Table 1:** Sequences predicted to be antigenic and linear B-cell epitopes in the studied hantavirus GP.

Hantavirus	Sequence	N of Predicted Algorithms	Vaxijen Score
SNV	71-PATTTQKYNQVDWTKKSSTTESTNAGATTFEAKTKE-106	6	1.16
	121-EAAYKSRKT-129	4	1.36
	281-PRGEDHDPDQNG-292	4	0.51
	302-ITAKVPSTETTET-314	5	0.59
	685-SSSSYSYRRKL-695	5	0.57
	920-MMATRDSFQ-928	4	0.64
	962-DVSFQDLSDNP-972	4	1.35
	1060-AASPPHLDRVT-1070	5	0.52
ANDV	71-TSMAQKSFT-79	6	0.6
	82-EWRKKSDTTDTTNAASTTFEAQTK-105	5	0.89
	682-LPSSSSYSYRRKLTNPA-698	5	0.46
SEOV	164-IQVVYERTY-172	5	0.65
	229-GSKCNNTDTKVQ-240	6	0.95
	765-PPDCPGVGT-773	5	0.93
	933-TDERIEWRDPDGM-945	3	0.89
	1048-ECSSTGLQASAPH-1060	5	1.23
PUUV	377-PGEIEKTTQ-385	5	0.96
	748-SCEKYAYPWQ-757	5	0.45
	775-CNPPDCPGVGTG-786	4	0.58
	947-SLEWIDPDSSLRDHI-961	5	0.74

**Table 2:** List of vaccine epitopes and their compositions.

Epitope	SNV_(84–__111)_	SNV_(116–134)_	SNV_(271–292)_
Sequence	TKKSSTTESTNAGATTFEAKTKEVNLKG	PPTTFEAAYKSRKTVICYD	AELFTRMVLNPRGEDHDPDQNG
B-cell	TKKSSTTESTNAGATTFEAKTKE	EAAYKSRKT	PRGEDHDPDQNG
T-cell	ATTFEAKTKEVNLKG	FEAAYKSRKTVICYD	AELFTRMVLNPRGED
T-cell	AGATTFEAK	PPTTFEAAY	-
T-cell	ATTFEAKTK	-	-
**Epitope**	**ANDV_(80–105)_**	**SNV_(298–314)_**	**SNV_(680–695)_**
Sequence	QVEWRKKSDTTDTTNAASTTFEAQTK	IAGPITAKVPSTETTET	DFALASSSSYSYRRKL
B-cell	EWRKKSDTTDTTNAASTTFEAQTK	ITAKVPSTETTET	SSSSYSYRRKL
T-cell	QVEWRKKSDTTDTTN	AGPITAKVPSTETTE	DFALASSSSYSYRRK
T-cell	DTTDTTNAA	IAGPITAKV	-
T-cell	NAASTTFEA	-	-
T-cell	STTFEAQTK	-	-
**Epitope**	**ANDV_(682–700)_**	**SNV_(914–928)_**	**SNV_(1060–1070)_**
Sequence	LPSSSSYSYRRKLTNPANK	VSGFQRMMATRDSFQ	AASPPHLDRVT
B-cell	LPSSSSYSYRRKLTNPA	MMATRDSFQ	AASPPHLDRVT
T-cell	LPSSSSYSYRRKLTN	VSGFQRMMATRDSFQ	ASPPHLDRV
T-cell	RKLTNPANK	-	-
**Epitope**	**PUUV_(940–967)_**	**SEOV_(160–172)_**	**SEOV_(229–246)_**
Sequence	EPHISASSLEWIDPDSSLRDHINVIVGR	GPYRIQVVYERTY	GSKCNNTDTKVQGYYICI
B-cell	SLEWIDPDSSLRDHI	IQVVYERTY	GSKCNNTDTKVQ
T-cell	EPHISASSLEWIDPD	GPYRIQVVY	SKCNNTDTKVQGYYI
T-cell	ASSLEWIDPDSSLRD	IQVVYERTY	KVQGYYICI
T-cell	DHINVIVGR	-	-
**Epitope**	**SEOV_(929–945)_**	**SEOV_(1045–1063)_**	**PUUV_(365–385)_**
Sequence	NIHFTDERIEWRDPDGM	HGKECSSTGLQASAPHLDK	TLPLTWTGFIPLPGEIEKTTQ
B-cell	TDERIEWRDPDGM	ECSSTGLQASAPH	PGEIEKTTQ
T-cell	NIHFTDERIEWRDPD	HGKECSSTGLQASAP	TLPLTWTGFIPLPGE
T-cell	FTDERIEWR	QASAPHLDK	-
**Epitope**	**PUUV_(740–757)_**		
Sequence	KTAFHCYGSCEKYAYPWQ		
B-cell	SCEKYAYPWQ		
T-cell	KTAFHCYGSCEKYAY		

**Table 3:** Assessment of potential allergenicity and toxicity of predicted sequences. Epitopes predicted to be allergenic or toxic were signalized with an asterisk (*).

Hantavirus	Epitope	AllergenFP	ToxinPred	HemoPI
SNV	SNV_(84–11)_	Non-allergenic	Non-toxic	Non-hemotoxic
	SNV_(116–134)_	Non-allergenic	Non-toxic	Non-hemotoxic
	SNV_(271–292)_	Non-allergenic	Non-toxic	Non-hemotoxic
	SNV_(298–314)_	Non-allergenic	Non-toxic	Non-hemotoxic
	SNV_(680–695)_	Non-allergenic	Non-toxic	Non-hemotoxic
	SNV_(914–928)_ *	Allergenic	Non-toxic	Non-hemotoxic
	SNV_(1060–1070)_ *	Allergenic	Non-toxic	Non-hemotoxic
ANDV	ANDV_(80–105)_	Non-allergenic	Non-toxic	Non-hemotoxic
	ANDV_(682–700)_	Non-allergenic	Non-toxic	Non-hemotoxic
SEOV	SEOV_(160–172)_ *	Allergenic	Non-toxic	Non-hemotoxic
	SEOV_(229–246)_	Non-allergenic	Non-toxic	Non-hemotoxic
	SEOV_(929–945)_	Non-allergenic	Non-toxic	Non-hemotoxic
	SEOV_(1045–1063)_ *	Allergenic	Non-toxic	Non-hemotoxic
PUUV	PUUV_(365–385)_	Non-allergenic	Non-toxic	Non-hemotoxic
	PUUV_(740–757)_ *	Non-allergenic	Toxin	Non-hemotoxic
	PUUV_(940–967)_	Non-allergenic	Non-toxic	Non-hemotoxic

**Table 4:** Comparison of vaccine sequence identities between hantavirus associated with HPS and HFRS. Values indicate the level of conservation, ranked by color as high (>70%, green), moderate (50% > X ≤ 70%, yellow), and low (≤50%, blue). Letters indicate amino acid residues, according to the one-letter code, differences in residue sequences are signalized by the respective changed letter, while conserved residues are indicated by *.

Related Disease	Hantavirus	SNV_(84–__111)_		SNV_(116–134)_	
		TKKSSTTESTNAGATTFEAKTKEVNLKG	Identity	PPTTFEAAYKSRKTVICYD	Identity
HPS	ANDV	R***D**DT***AS*****Q**T***R*	67.86%	A*ELYDTLK*VK***L***	42.11%
	SNV	TKKSSTTESTNAGATTFEAKTKEVNLKG	100.00%	*******************	100.00%
	LAGNV	R**AE**DT*Q*AS*****Q**T**IR*	57.14%	ASDLYDTLKRVK***L***	31.58%
	CHOV	A***T***T**********TS*ST**R*	71.43%	SSDL*DTYK*VK***L***	42.11%
	RIOMV	R***E**DT*E*A******Q**TA**R*	64.29%	A*DLYDTLKRIK***L***	36.84%
HFRS	SEOV	VIWRKKANQES*NQNS**VVES**SF**	25.00%	KHRMV*ES*RN*RS*****	42.11%
	PUUV	EM*VDLA*N*Q*SS*S*QT*SS*I**R*	39.29%	**LVI*T*ART***IA*F*	47.37%
	HANTV	VIWRKKANQES*NQNS**VVES**SF**	25.00%	KHRMV*ES*RN*RS*****	42.11%
	DOBV	VSWRKKADKAQ*AKDS**TTSS******	32.14%	SHRMV*ES*RN*RS*****	42.11%
	TULAV	E**AD*A*NAK*AS***QSSS***Q*R*	46.43%	*TLVL*T*SRT****T*F*	47.37%
	THAIV	VIWRKKADQAS*NQNS**VVES*ISF**	21.43%	KHRMI*ES**N*RS*****	47.37%
**Related Disease**	**Hantavirus**	**SNV**_(271–292)_		**SNV**_(298–314)_	
		AELFTRMVLNPRGEDHDPDQNG	**Identity**	IAGPITAKVPSTETTET	**Identity**
HPS	ANDV	**VVS**LVH*******AI**S	59.09%	*V**********SS*D*	76.47%
	SNV	**********************	100.00%	*****************	100.00%
	LAGNV	***AS**L*H********L**A	72.73%	*V*********SSS***	76.47%
	CHOV	**IVS*IIMH*********KA*	59.09%	*****K****Q***S**	82.35%
	RIOMV	**VVS**IVH********L**A	63.64%	*V**V*******SSAD*	64.71%
HFRS	SEOV	M*V*SGIITS*H*****LPGEE	36.36%	*S*Q*E**I*H*VSSKN	41.18%
	PUUV	**VLS**AFA*H*****IEK*A	50.00%	*V*KV*G*A****SSD*	52.94%
	HANTV	M*V*SGIITS*H*****LPGEE	36.36%	*S*Q*E**I*H*VSSKN	41.18%
	DOBV	M*ALASLLRA*H*****LSGEE	31.82%	***Q*EG*I*H*ANAAN	41.18%
	TULAV	S*ILS**TTA*H*****I*P*A	50.00%	*V*QL*G*A****SSD*	52.94%
	THAIV	M*I*SNIL*S*F*****LPGEE	40.91%	*****E**I*H*VSSKN	52.94%
**Relate Disease**	**Hantavirus**	**SNV**_(680–695)_			
		DFALASSSSYSYRRKL	**Identity**		
HPS	ANDV	**S*P***********	87.50%		
	SNV	****************	100.00%		
	LAGNV	**S*P*********R*	81.25%		
	CHOV	**S*P***********	87.50%		
	RIOMV	**S*P***********	87.50%		
HFRS	SEOV	**S*P***K*T*K*H*	62.50%		
	PUUV	**S*P**A**T***Q*	68.75%		
	HANTV	**S*P***K*T*K*H*	62.50%		
	DOBV	**S*P***K*T*K***	68.75%		
	TULAV	**S*P**AT*T***E*	62.50%		
	THAIV	**S*P***K*T*K***	68.75%		
**R** **elated Disease**	**Hantavirus**	**ANDV _(80–105)_**	**ANDV_(__682–700)_**		
		**QVEWRKKSDTTDTTNAASTTFEAQTK**	**Identity**	**LPSSSSYSYRRKLTNPANK**	**Identity**
HPS	ANDV	**************************	100.00%	*******************	100.00%
	SNV	**D*T***S**ES***GA*****K**	69.23%	*A***********V****Q	84.21%
	LAGNV	*L*****AE*****Q***********	84.62%	***********R*******	94.74%
	CHOV	**N*A***T**E****GA*****TS*	69.23%	*************I****H	89.47%
	RIOMV	********E*****E**A********	88.46%	*******************	100.00%
HFRS	SEOV	*KGNTY*IV*AI*SAMG*KCNNTD**	26.92%	*****K*T*K*H****V*D	68.42%
	PUUV	KWT*EM*V*LAEN*Q*S**S*QTKSS	30.77%	****A**T***Q*Q****E	73.68%
	HANTV	*KGNTY*IV*AI*SAMG*KCNNTD**	26.92%	*****K*T*K*H****V*D	68.42%
	DOBV	K*S****A*KAQAAKDSFE*TSSEVN	26.92%	*****K*T*K****S*L*Q	68.42%
	TULAV	KWS*E**A**AENAK******QSSS*	46.15%	****AT*T***E*Q****E	68.42%
	THAIV	K*I****A*QASANQNSFEVV*SEIS	26.92%	*****K*T*K******I*V	73.68%
**Related** **disease**	**Hantavirus**	**SEOV**_(229–246)_	**SEOV**_(929–945)_		
		GSKCNNTDTKVQGYYICI	**Identity**	NIHFTDERIEWRDPDGM	**Identity**
HPS	ANDV	LT*TGC*ENAL****V*F	38.89%	EP*I*TNKL**I****N	47.06%
	SNV	ITGVSC*ENSF******F	38.89%	EP*I*SN*L**I***SS	47.06%
	LAGNV	I**TGC*ENSI****V*F	44.44%	DP*I*SSKL**I****N	47.06%
	CHOV	LQ*NDC*TNNF**F*V*F	33.33%	*P*L*ANNL**T****A	52.94%
	RIOMV	I**TGC**NAI****V*F	50.00%	DP*I*TN*L**I****N	52.94%
HFRS	SEOV	******************	100.00%	*****************	100.00%
	PUUV	VQ*TGC*GNGF****V*L	38.89%	EP*ISASSL**I***SS	35.29%
	HANTV	******************	100.00%	*****************	100.00%
	DOBV	ANN**D**N******L**	66.67%	S**Y*******K*****	82.35%
	TULAV	VE*TGC*ENALK***A**	38.89%	EP*I*TNSL**V***SS	41.18%
	THAIV	NN***D*EM*********	72.22%	S****************	94.12%
**Related Disease**	**Hantavirus**	**PUUV**_(365–385)_	**PUUV**_(940–967)_		
		TLPLTWTGFIPLPGEIEKTTQ	**Identity**	EPHISASSLEWIDPDSSLRDHINVIVGR	**Identity**
HPS	ANDV	*V*I****YL*IS**M**V*G	57.14%	****TTNK*******GNT***V*LVLN*	57.14%
	SNV	*V*******LAVS*****I*G	66.67%	****TSNR*********IK****MVLN*	64.29%
	LAGNV	********YL*IS**M**V*G	66.67%	D***TS*K*******GNT***V*L*LN*	60.71%
	CHOV	********YL*IS**M**I*G	66.67%	N**LT*NN***T***GATK**V*LVLN*	46.43%
	RIOMV	*I******YL*IS**M**V*G	61.90%	D***TTNR*******GNT***V*LVLN*	53.57%
HFRS	SEOV	A***I*R*L*D*T*YY*AVHP	42.86%	NI*FTDERI**R***GM******IVISK	42.86%
	PUUV	*********************	100.00%	****************************	100.00%
	HANTV	A***I*R*L*D*T*YY*AVHP	42.86%	NI*FTDERI**R***GM******IVISK	42.86%
	DOBV	A***I*E*Y*D***YY*TVHP	47.62%	SI*YTDERI**K***GM*K**L*IL*TK	39.29%
	TULAV	**********T**********	95.24%	****TTN****V******K**V*L**N*	71.43%
	THAIV	A**VV*R*M*D*S*YY*AVHP	38.10%	SI*FTDERI**R***GM******IVITK	42.86%

**Table 5:** Physicochemical features of the multi-epitope vaccine constructs. MEB: multi-epitope base, VC1: Vaccine Construct 1; VC2: Vaccine Construct 2.

Physicochemical Feature	Reference Value	MEB	VC1	VC2
Adjuvant	N.A.	None	β-defensin	50Srp
Molecular weight (Da)	N.A.	29,112.18	34,712.91	42,934.09
Instability index	<40	29.54	30.72	26.16
		(stable)	(stable)	(stable)
Gravy score	<0	−0.703	−0.685	−0.422
Estimated half-life (hours)				
Mammalian reticulocyte	>10	7.2	30	30
Yeast		>20	>20	>20
*E. coli*		>10	>10	>10
Aliphatic index	>50	53.76	52.99	69.78
Allergenicity	Non-allergenic	Non-allergenic	Non-allergenic	Non-allergenic
Antigenicity score	>0.4	0.737	0.712	0.618

**Table 6:** Molecular docking details of proposed vaccines and immune receptor complexes.

Tool	Parameter	VC1		VC2	
		TLR2	TLR4	TLR2	TLR4
ClusPro	Members (n)	173	68	82	74
	Weighted score—Center	−1580.1	−1352.9	−1464.3	−1507.2
	Weighted score—Lowest energy	−1889.6	−1421.7	−1656.2	−1746.4
Prodigy	ΔG (kcal mol-1)	−13.7	−12.3	−14	−15.6
	Kd (M) at 37 °C	2. 1 × 10^−10^	2.2 × 10^−9^	1.5 × 10^−10^	9.3 × 10^−12^
CoCoMaps	Interacting Residues (n)	68	53	73	84
	Interacting Residues in TLR (n)	34	33	37	47
	Interacting Residues in VC (n)	34	20	36	37
	Number of H-bonds	11	12	8	13
	Number of CH-O/N bonds	10	11	11	22
	Number of Apolar vdW contacts	20	7	20	17
	Number of Salt-bridges	5	3	5	9
	BSA (Å^2^)	2962.4	1888.8	2854.8	3162.6
	Interface area (Å^2^)	1481.2	944.4	1427.4	1581.3
	Buried area (%)	7.1	4.51	6.55	7.14
	POLAR BSA (Å^2^)	1183.6	793.2	997.3	1418.7
	POLAR Interface area (Å^2^)	591.8	396.6	498.65	709.35
	POLAR Interface (%)	39.95	41.99	34.93	44.86
	NON POLAR BSA (Å^2^)	1778.8	1095.6	1857.5	1744.1
	NON POLAR Interface area (Å^2^)	889.4	547.8	928.75	872.05
	NON POLAR Interface (%)	60.05	58.01	65.07	55.15

## Data Availability

The original contributions presented in this study are included in the article/Supplementary Material. Further inquiries can be directed to the corresponding authors.

## Supplementary Materials

The following supporting information can be downloaded at https://www.mdpi.com/article/10.3390/ijms27157021/s1.
