# Supplementary material for: Broad-Spectrum Multi-Epitope Design Targeting Conserved Hantavirus Glycoproteins (Gn/Gc): Chimeric Antigen Engineering and Structural Mapping
Source: Int J Mol Sci. 2026 Aug 5;27(15):7021. doi: 10.3390/ijms27157021 (PMC13467019; doi:10.3390/ijms27157021)
Supplement: Supplementary file 1 [file ijms-27-07021-s001.zip › Figure_S1.pdf]

MolProbity Ramachandran analysis

vac1-escolhido_2_1_trimmed.pdb, model 1

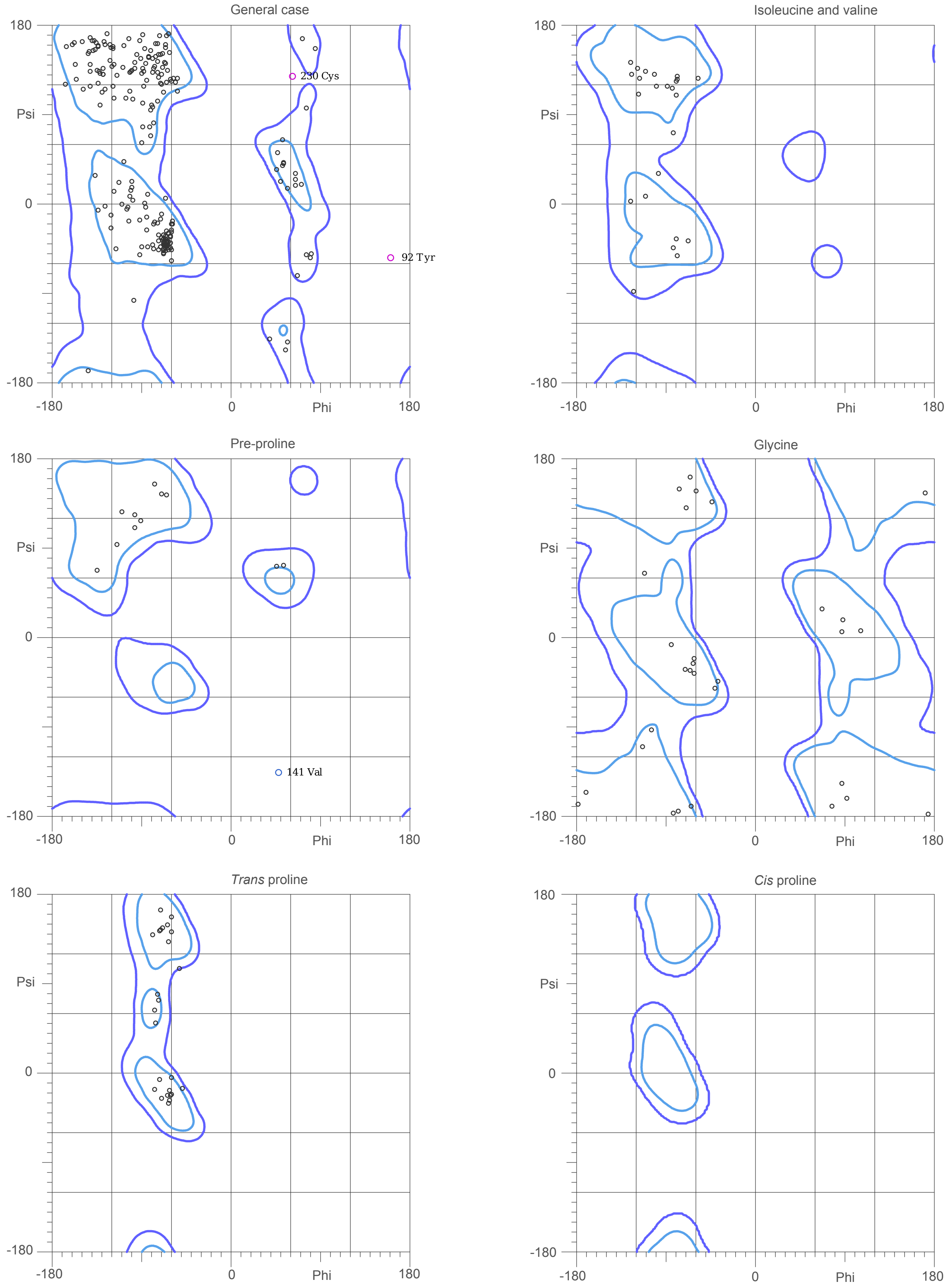

91.2% (291/319) of all residues were in favored (98%) regions.
99.1% (316/319) of all residues were in allowed (>99.8%) regions.

There were 3 outliers (phi, psi):
92 Tyr (161.5, -54.5)
141 Val (48.2, -136.5)
230 Cys (62.8, 129.7)

http://kinemage.biochem.duke.edu

Lovell, Davis, et al. Proteins 50:437 (2003)
