# Supplementary material for: Broad-Spectrum Multi-Epitope Design Targeting Conserved Hantavirus Glycoproteins (Gn/Gc): Chimeric Antigen Engineering and Structural Mapping
Source: Int J Mol Sci. 2026 Aug 5;27(15):7021. doi: 10.3390/ijms27157021 (PMC13467019; doi:10.3390/ijms27157021)
Supplement: Supplementary file 1 [file ijms-27-07021-s001.zip › Figure_S2.pdf]

MolProbity Ramachandran analysis

vac2-escolhidopdb_3_1_trimmed.pdb, model 1

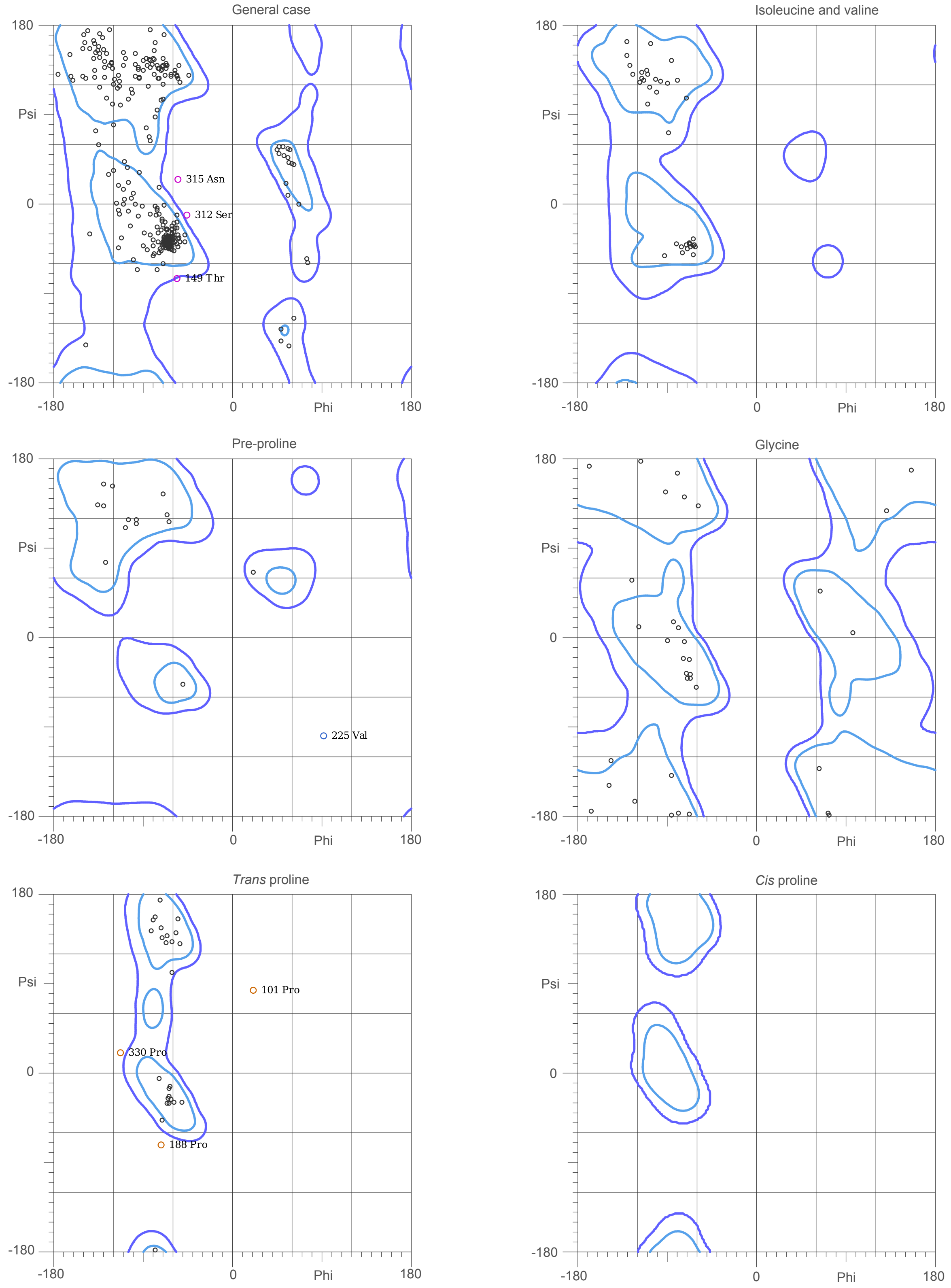

91.8% (370/403) of all residues were in favored (98%) regions.
98.3% (396/403) of all residues were in allowed (>99.8%) regions.

There were 7 outliers (phi, psi):
- 101 Pro (21.7, 84.2)
- 149 Thr (-56.6, -75.5)
- 188 Pro (-72.4, -72.7)
- 225 Val (93.0, -99.5)
- 312 Ser (-46.4, -11.3)
- 315 Asn (-55.9, 25.1)
- 330 Pro (-113.0, 21.6)

http://kinemage.biochem.duke.edu

Lovell, Davis, et al. Proteins 50:437 (2003)
